# Canadian Career Firefighters’ Mental Health Impacts and Priorities

**DOI:** 10.3390/ijerph182312666

**Published:** 2021-12-01

**Authors:** Joy C. MacDermid, Margaret Lomotan, Mostin A. Hu

**Affiliations:** 1 Physical Therapy and Surgery, Western University, London, ON N6G 1H1, Canada; 2 Clinical Research Lab, Hand and Upper Limb Centre, St. Joseph’s Health Centre, London, ON N6A 4V2, Canada; 3 Rehabilitation Science, McMaster University, Hamilton, ON L8S 1C7, Canada; lomotam@mcmaster.ca; 4 Clinical Medicine, University of Cambridge, Cambridge CB2 0SP, UK; mah240@cam.ac.uk

**Keywords:** firefighters, mental health, occupational stress injury

## Abstract

Firefighters’ perceptions of mental health can inform management. This qualitative study explored Canadian career firefighters’ experiences, needs, and research priorities with respect to mental health. Thirty-nine career firefighters (33 men, 6 women) of different ranks and geographic locales were interviewed using a semi-structured interview guide. The interviews were recorded, transcribed, and qualitatively analyzed using thematic analysis within an interpretive description approach. Firefighters reported that critical incidents and chronic job stressors contributed to mental health symptoms that led to burnout, compassion fatigue, and mental and physical injury. They were concerned with family impacts, like lack of full openness, reduced financial stability, and risk of divorce; and work impacts, like interpersonal conflict, lack of support to fellow firefighters, task avoidance, and absenteeism. A broad array of barriers and facilitators were found in firefighter work, culture, programs, social supports, health care, and societal factors. Variability in access to help, the changing fire service, and the complexity of knowing what to do to achieve mental health were evident across themes. Firefighters identified the need for research in four areas: awareness and monitoring, understanding etiology of mental health, better prevention and treatment, and access to care. Across domains of inquiry, context, “two sides to the coin”, and uncertainty were overarching themes.

## 1. Introduction

Firefighters are exposed to high rates of traumatic events, such as being assaulted in the line of duty and death/severe injury of victims or fellow firefighters [1,2,3]. A study of Canadian and American firefighters indicates most had exposure to at least one such event in the prior year—many of which involved encountering death by suicide or graphic, deadly tragedies [4]. The five most common critical incidents and distressing exposures were seeing someone die, a recently dead body, badly beaten adult, severely neglected child, or completing a death notification [5]. Recent studies of individual fire services and a national survey confirm high rates of critical event exposures in Canadian firefighters [6,7]. We found that 64% of career firefighters had been exposed to a death in the line of duty in the past year, with 14% of these cases involving the death of a child. Further, 25% had required police protection while completing their work. Since women firefighters are underrepresented in any one fire service, we sampled nationally and found similar exposure rates for women and men firefighters [7]. In Canada, smaller communities tend to be serviced by volunteer firefighters. Brazil found 92% of volunteer firefighters from one province in Canada had been exposed to a critical event, and 35% had been exposed to more than 20 events [8]. Of concern, only 3% had received individual debriefing. This suggests that the volunteer firefighters may have different needs and priorities than career firefighters.

It is well known that occupational stress injury occurs in public safety personnel (PSP) at much higher rates due to the high rates of critical events exposures. Post-traumatic stress disorder (PTSD) is one potential outcome from critical event exposures [9], as are major depressive or panic disorders [2,10,11,12]. National data show that 14–33% of PSP report symptoms consistent with the diagnostic criteria for PTSD and major depressive disorders [13] and that this is associated with elevated suicidal ideation and attempts [14]. The nature of firefighting work and male-gender expectations can both contribute to unresolved PTSD and depressive symptoms, which are associated with family problems [15,16], alcoholism, and drug addictions [17,18]. This may explain the elevated suicides rates amongst firefighters [19,20,21].

Firefighters share many exposures with other PSP, especially in terms of stress exposures. However, firefighters differ in terms of their exposure in comparison to other PSP depending on the type of firefighting work required and the nature of the communities they serve. Firefighters have periods of high stress work that maximally tax their mental, cardiovascular, and musculoskeletal (MSK) systems. Their work involves risky work tasks, 24-h shifts, rapid changes from rest to emergency response, infectious/chemical hazards, and unstable, unfamiliar work zones. Firefighters incur high rates of traumatic falls, burns, crush injuries [22,23,24], chronic MSK disorders, occupational diseases (cancers, respiratory diseases), and PTSD [25,26,27,28,29,30,31,32]. While mental and physical health are often separated, there is growing realization that this is an artificial divide since MSK disorders and mental health problems are often associated [19], with stress theorized as a common mechanism [33]. Physiological response to the physical demands, injuries, or cancers that arise due to toxic exposures are exacerbated by mental health issues [34]. Diagnosis and management of specific disorders, like PTSD, and management of generic mental stress are both needed to ensure overall health and work ability in firefighters.

Public policy, clinical innovation, and local investment in programs and research priorities are all informed by understanding the priorities, experiences, and needs of PSP. In response to research documenting the high rates of mental health concerns amongst Canadian PSP [13,14], the Canadian government developed “*Supporting Canada’s Public Safety Personnel: An Action Plan on Post-Traumatic Stress Injuries*” [35]. This Action Plan invested $20 million over five years in research to improve risk mitigation, diagnosis, and treatment and supported a new national research consortium between the Canadian Institutes for Health Research (CIHR) and the Canadian Institute for Public Safety Research and Treatment (CIPSRT). This was designed as a coordinated research plan with three pillars: (1) Research and Data Collection; (2) Prevention, Early Intervention, and Stigma Reduction; and (3) Support for Care and Treatment. These pillars confirm that research priorities must be linked to needs and priorities while advancing the science that underpins the way these are addressed. Twenty-two one-year catalyst grants on post-traumatic stress injuries among PSP were funded, followed by larger four-year team grants—for a subset of the phase I applicants—that supported mental wellness in public safety projects. Thus, the topics funded were largely driven by the expertise, relationships, and research questions posed by the research applicants that applied to that targeted competition.

In some jurisdictions, more formal priority setting of research questions has been undertaken. The National Fallen Firefighters Foundation (NFFF) has hosted multiple research agenda symposiums with results published from the consensus exercises completed in 2005 and 2011 (https://www.everyonegoeshome.com/16-initiatives/7-research-agenda/ (accessed on 30 November 2021)). The National Fire Service Research Agenda was intended to identify research areas that would contribute to the Firefighter Life Safety Initiatives of the Everyone Goes Home Program^®^. The resulting agenda was intended to assist the many different organizations, researchers, or individuals involved in conducting, supporting, and encouraging research. The intention was to help direct applied research into areas that have been identified as important and significant by firefighter stakeholders, with the overarching goal of eliminating preventable firefighter line-of-duty injuries and fatalities. The latest National Fire Service Research Agenda Symposium was held 15 February–10 March 2021 and will lead to an updated research agenda. Although it did not include a research agenda, the 2021 Canadian Association of Fire Chiefs (CAFC) report, Health and Wellness: Taking Care of the Front Line, Mental Health, and Musculoskeletal Injuries, addressed issues in these two key areas of firefighter health.

A substantial body of literature is available on firefighter health. However, much of it is epidemiological studies documenting the nature of their exposures and injuries, factors predicting injury, or responses to specific treatment programs. These approaches provide empirical data on burden of illness that are important considerations in program and policy design and setting research priorities. Qualitative studies that help us to understand burden, needs, and priorities are infrequently reported. A recent narrative review summarized studies—mostly quantitative—on fire culture, treatment barriers, practice implications, and research directions [36]. The review highlighted the concerns about the rate of mental health disorders and suicides. It noted that there was little data regarding the utilization of health services by firefighters. However, limited data suggested that those with more severe mental illness tended to seek treatment at some point in their career, but barriers to accessing care were also noted. This included stigma, concerns about reputation, and structural barriers, like cost and time missed from work. These problems limited the impact of potential treatments in real-world effectiveness. Suggestions for future research were based on the analysis of gaps noted in the literature but did not involve direct consultation with firefighters. The authors concluded that research was needed to address stressors and mental health and risk factors and study long-term treatment outcomes in currently used interventions.

Although quantitative studies have established that mental health should be a priority given high risks of mental health problems and addictions in firefighters, there has been little qualitative research to explore firefighters’ perceptions in relation to the problem. Grassroots input is important. Therefore, the purposes of this study were to explore Canadian career firefighters’ perceptions with respect to the impact of mental health stressors on their health, family, and work; their perceived barriers and facilitators to maintaining their mental health wellness; and ideas about how research could improve health and wellness for firefighters.

## 2. Methods

### 2.1. Study Approach/Design

Interpretive description is a method that draws on the traditions and approaches of multiple traditional forms of qualitative inquiry. It arose in nursing to “contribute directly to our understanding of how people experience their health and illness and what nursing can do to make a difference” [37]. It has since diffused into other fields that share similar goals in qualitative inquiry. Interpretive description was selected as the ideal approach for this study since it is an inductive approach that focuses on capturing themes and patterns within subjective perceptions and generating an interpretive description capable of informing a practically useful understanding [37,38,39,40]. This aligns with our purpose to understand career firefighters’ experiences and needs with respect to mental health and what research or programs make a difference. Further, interpretive description relies on previous theoretical or practical knowledge and expects authors to be embedded within the topic and co-create the knowledge with participants rather than act as independent observers. This is consistent with the long-standing relationship between the researchers and the firefighter community.

### 2.2. Sampling, Recruitment, and Consent of Participants

Purposeful sampling was constructed to focus on career firefighters and to obtain the perceptions of firefighters in different ranks varying from front-line firefighters to fire chiefs. We purposely sampled a variety of geographical locations to have variance in the nature of the community being served and firefighter roles performed, and we ensured that women firefighters were included. This study protocol was approved by the Hamilton Integrated Research Ethics Board (Project Number #3289, Date of Approval: 3 May 2017). Informed consent was obtained from all subjects involved in the study. Due to concerns about anonymity, we did not keep records on personal factors, like age, and did not associate personal factors with quotes.

### 2.3. Role of the Researchers

Interpretive description recognizes that knowledge is co-created by participants and researchers since the practical purpose drives the goals, methods, and interpretation of the work. The first author is a physical therapist and epidemiologist researcher with more than 15 years of experience working with firefighters in co-designed research and educational activities that address mental and physical health. The second author is a research associate who has worked with firefighters on research and knowledge translation activities for more than 10 years. The third author was an independent student.

### 2.4. Data Collection Procedures

A semi-structured interview guide was designed based on the research questions and was discussed by study authors prior to implementation. Interviewees were provided options on timing of interviews and interviewed in-person or by telephone. Given the national focus, most interviews (*n* = 38) were conducted remotely. Two experienced interviewers conducted the interviews by asking all major questions and exploring issues that needed clarification or where the participant wished to add additional information based on a semi-structured interview guide. The interview guide was designed to explore sequential pathway of topics related to the experiences and needs of firefighters (See Supplementary File S1). The firefighters (33 men, 6 women) included senior officers: Lieutenant/Acting Captain/Captain (*n* = 11) and Assistant Deputy Chief/Deputy Chief/Chief (*n* = 7). The participants were from: British Columbia (*n* = 2), Alberta (*n* = 1), Nunavut (*n* = 2), Quebec (*n* = 6), and Ontario (*n* = 28).

### 2.5. Analysis

In keeping with the intention to understand the characteristics and patterns within firefighter mental health, the recorded interviews were transcribed into typewritten text and qualitatively analyzed using thematic analysis within an interpretive description approach. Thematic analysis was used to identify, analyze, and report patterns or themes using the specific categories of inquiry identified as the practical issues that need to be delineated for our study purpose. The analysis followed the six phases of thematic analysis described by Braun and Clarke (familiarizing with data, generating initial codes, searching for themes, reviewing themes, defining and naming themes, and producing the report) [41]. In keeping with interpretive description, repeated immersion in the data was used to drive coding, classifying, and linkages rather than line-by-line coding. Fieldnotes from the data collection and from forums where the topic was discussed within the firefighter environment and associated conferences/meetings were collected and used for reflection on the emerging themes.

The transcribed interviews were reviewed by two researchers who independently coded the interview transcripts. Researchers assigned text for coding, identified quotes associated with the codes from the transcripts, and developed codes into themes. The independently developed codes and themes were discussed with team members as needed.

### 2.6. Trustworthiness of Findings

To verify our data, we used the following: concurrent data collection and analysis, constant comparative analysis, and iterative analysis, locating explanatory factors within the larger perspective. To improve trustworthiness, we used multiple coders and formally reflected on the authors’ experiences/opinions and how they relate to the study questions. The findings were presented to multiple groups of firefighters and researchers to assess questions and feedback before finalizing the paper. Any identified gaps/differences were discussed amongst the researchers until consensus was obtained.

## 3. Results

**Figure 1:**
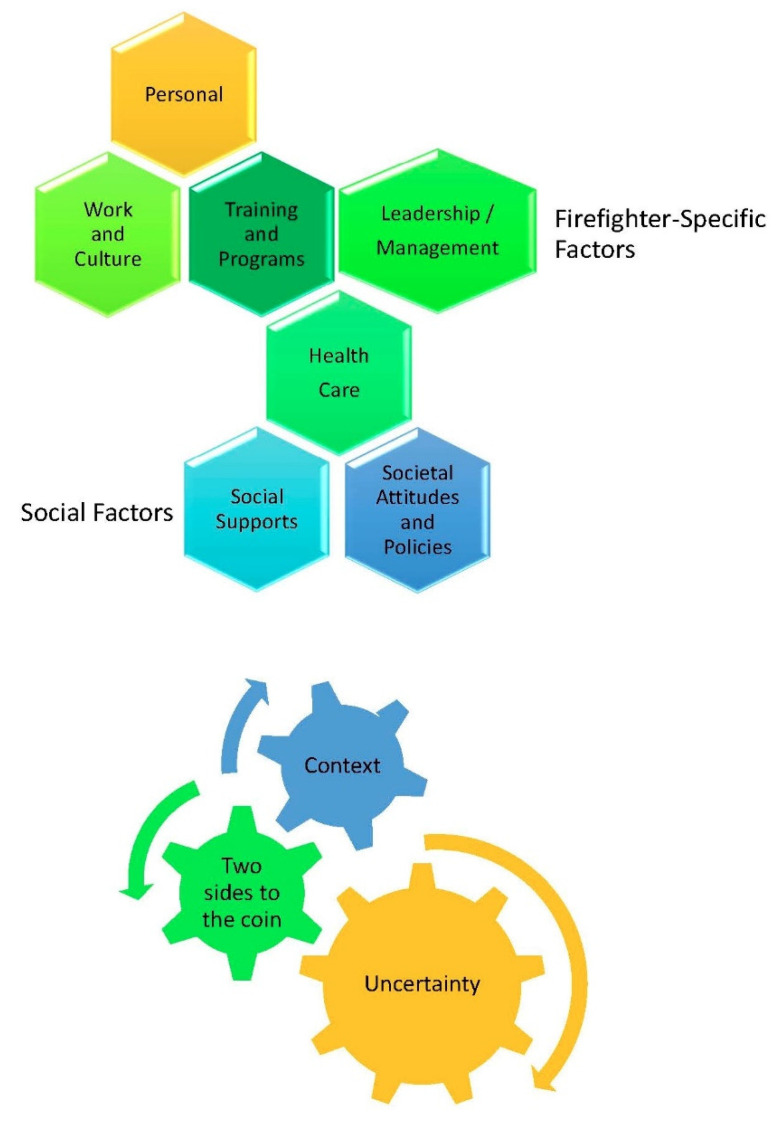
Firefighter Mental Health Themes.

The results are organized to address the major questions and themes that emerged on the impacts of mental health (personal, family, and work) and associated barriers and facilitators (personal, fire work and culture, fire service training and programs, leadership and management, health care, social supports, and societal attitudes and policies). Although firefighters were uncertain about identifying research priorities, their responses reflected these themes and the needed types of research. The overarching latent themes that arose across all lines of inquiry were the importance of contextual differences, that issues had “two sides to the coin”, and that there is a great deal of uncertainty in mental health (Figure 1). This was reflected in differences in needs, experiences, stressful incidents, response to incidents, access to help, and mental health outcomes.

Several firefighters expressed that they preferred the use of the term occupational stress injury (OSI) rather than other terms that are used to describe mental health problems that arise in PSP. While they acknowledge that recent increased emphasis on PTSD was positive in many ways, they also acknowledged the other side of the coin was that sometimes the focus on this singular diagnosis takes away from their broader concerns around mental health:

“*There is more than just PTSD out there. PTSD is a bit of a fail. We’ve missed the opportunities to help people along the way.*”

### 3.1. Impacts

**Figure 2:**
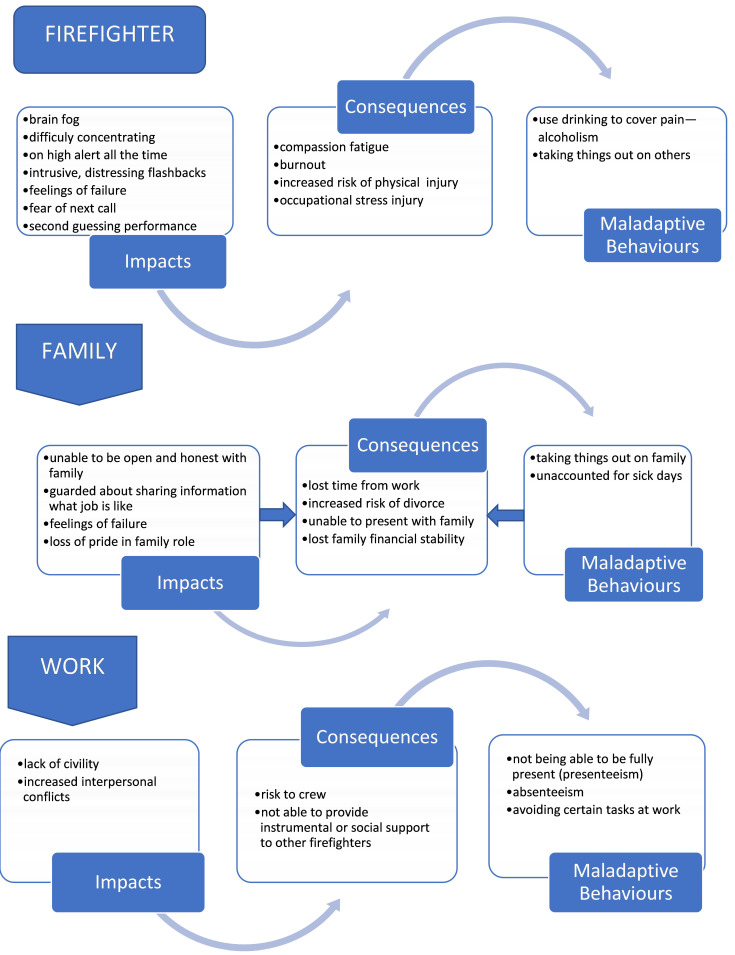
Impacts, Consequences, and Maladaptive Behaviours due to Occupational Stress.

Firefighters noted challenges in maintaining mental health throughout their career and that both individual critical incidents and sustained stress over time contribute to the problems that they experience. Therefore, acknowledging both was important. Firefighters reported a variety of symptoms arising from occupational stress. Broadly, these themes focused on the impacts—either directly on the firefighter or indirectly on the family or the work, with trickle-down consequences and concerns about maladaptive behaviours or limited coping (Figure 2).

“*Someone in my office today that is off on post-traumatic stress disorder that is not recognized by the Workman’s Compensation Board, and he can’t sleep, doesn’t want to go on medical calls anymore, he has nightmares about coming back to work. He’s starting to drink, there’s substance abuse, there’s relationships issues, there’s divorces, impacts on the kids. It’s really large.*”

#### 3.1.1. Impacts on Firefighters

Classic symptoms of mental health overload were evident in the symptoms reported: brain fog, difficulty concentrating, on high alert all the time, intrusive and distressing flashbacks, feelings of failure, fear of the next call, and second guessing your performance.

“*I’ll give you an example. I came in on a person—she had hung herself, and I can still see her perfectly straight black hair, I can still see her pajamas, and I can still see her hanging there. And that was 15 years ago. It’s like I’m still standing there staring at her hanging from the ceiling. How does it affect you? I don’t know how it affects me, but it does. The memories stay.*”

Firefighters acknowledged that these feelings had consequences, and many struggled with the fact that, over time, they felt that they became burned out or developed compassion fatigue. They acknowledged that they had become hardened to many critical incidents and atrocious sights that were not “normal” for most of society. While acknowledging that these were in some ways requirements of the job and necessary to protect their own mental health, they also felt guilty about no longer having the same level of emotional response. They also acknowledged that despite feeling hardened to such sights, these stressors build up over time, which means it can be hard to identify at what point they would have an impact on their mental health. Despite feeling hardened, many also expressed second-guessing themselves:

“*Let’s go to the worst case scenario. You get there, and the whole family has passed away in the house fire. And what’ll happen is the firefighter will say, oh God, if we’d only got there, you know, one minute sooner, if I’d only taken this turn or that turn maybe we could have.*”

Another concern raised by firefighters was that mental health concerns, like difficulty concentrating or second-guessing yourself, can increase the risk of physical injury.

#### 3.1.2. Impacts on Firefighters’ Families

Many firefighters spoke about the impact on their families. While firefighters expressed that families were a major support and that they desired to have open communication with their spouses or other family members, the other side of the coin was that it was difficult to be honest with their family members about their daily experiences:

“*So, it’s difficult when you talk to your loved ones, it can be your friends that aren’t firefighters—it just becomes very difficult because they don’t really get it. They think you’re upset about something, but they don’t know why and it causes a lot of—it can cause a lot of grief.*”

“*I’ve seen a psychologist for PTSD because of the things that I’ve seen and done. And I can say that it’s affected my family, in a way, that I do drink too much at times. And I don’t take it out on my family, but you don’t want to share with them, so you do hold a lot of things in.*”

Reasons given were that they were concerned that their family member might change the way they viewed them as a person because of the kinds of things they were seeing at work. Concurrently, they also wanted their family members to have faith that their role as a firefighter was valuable and that their job was good and important, and they were worried that sharing some of their experiences might undermine their families’ valuation of the job. These difficulties in being open and transparent with family members contribute to why firefighters have such strong bonds with other firefighters since they are confident they can be open and transparent with other firefighters who share similar experiences:

“*Ideally, another firefighter because we’re—we’re a very kind of close-knit, closed group.*”

Firefighters acknowledged that the issues that impact their confidence in self and feelings of failure from bad outcomes at work carried over into their feelings within the family. Firefighters also acknowledged awareness and regret that mental health stressors from work carried over into their family life in terms of how they treated family members with less tolerance, irritability, or poor communication:

“*I could see something on a call, come home, have—put it in the back of my head for a couple of days, and then, you know, get into a fight with my spouse, and then all of a sudden, all that emotional weight from the call before comes out.*”

They were concerned that taking time off work often compromised their family’s financial stability since sick benefits do not replace usual firefighter income. Many acknowledged the increased risk of divorce in the fire service or had personally experienced divorce and attributed work issues as a contributing factor:

“*One’s very personal for me, being off on PTSD, it’s significant, it’s especially because you included families, that when I look at a divorce rate and the problems that we have at home, it’s just anecdotal, but I see them as higher.*”

#### 3.1.3. Impacts on Workplace

Firefighters also acknowledged that their mental health had an impact on the workplace, their fellow firefighters, and their ability to perform their firefighter role. They acknowledged that they themselves had behaved with less civility to their colleagues when they were experiencing mental health challenges and felt that they had observed similar trends in others:

“*I would say one of the things is interpersonal conflicts that can easily flare up due to stress.*”

“*… if you’re not dealing with it, you can get really bitter, really snappy with the guys.*”

They felt that sometimes they were not able to provide either instrumental or social support to their fellow firefighters because they were struggling with their own issues. They expressed concerns about not being fully present when at work (presenteeism) and increased rates of absenteeism when experiencing stress or mental health challenges.

“*Somebody has a very traumatic call, and they didn’t really have a good feeling about it, and then the next time they have an incident that’s quite similar to that, maybe it could bring back, you know, flashbacks and memories of the other one that kind of traumatized them, and it kind of take their focus away from doing their job safely and effectively.*”

They were concerned the impact that mental health had on their job performance, “*So, it affects the comradeship between the guys. It affects the level of service that we provide,*” and were particularly concerned that this placed additional risk or workload on their crew members. While they recognized the importance of time off for mental health, the other side of the coin was their concerns that there was potential for taking time off for mental health to be misinterpreted or misused and the impacts on the fire service:

“*You’re just not wanting to go to work because you don’t want to have another... maybe an infant call or, you know, another dead body or something. You don’t want to see that over and over again, so you maybe just book off sick, so that affects corporation, you know, overtime and everything else.*”

“*If I could go without, you know, serious repercussions, and, for instance, jump in a boat, and go clear my head fishing for the day, you just need to have the support from employers to go and clear your head, but it’s a fine line, because you don’t want to have people abuse it.*”

“*So, you become less of a team player, maybe not by choice but more by the effect of the mental strain or stress that you’re suffering from, from a critical incident or mental trauma. So those types of things definitely affect your firefighting.*”

### 3.2. Barriers and Facilitators

Firefighters explained an array of barriers and facilitators that affected their mental health. The themes that emerged focused on personal, fire service, and societal factors and revealed that while context was often a driver of what factors were most influential within a given fire service, it was also true that considerable individual diversity had to be accounted for since firefighters acknowledge that the same incident, program, treatment, or policy may be helpful to one firefighter and not helpful or even harmful to another (Figure 2).

Firefighters recognized “two sides to the coin” in themselves and the fire service, and this was clearly evident across different firefighters. That is, multiple personal or work factors were seen as positive or negative but also had the alternative side. The traumatic nature of their work is both a stressor but also contributes to their recognition of its importance and helps them appreciate the good in their own lives. A culture of toughness and dark humour was seen both as a valued coping strategy and a potential deterrent for some to disclose mental health concerns.

Although there was disparity across different jurisdictions, a substantial number of mental health programs were mentioned by firefighters as being positive. Many firefighters were aware of things already in place, including peer support, resiliency training, critical incident response, suicidal awareness programs, and PTSD prevention plan—there were positive comments about all of these programs. Resiliency programs that focus on increasing awareness of mental health stressors, coping skills, and recognizing signs of mental health problems in self or others, such as the Road to Mental Readiness or Resilient Minds^TM^ (https://resilientminds.cmha.ca/ (accessed on 30 November 2021)), were seen as highly beneficial by departments where they had been implemented and seen as highly desirable for those who had not yet received this training. However, unequal access was clear across fire services and a major contextual factor.

Some firefighters noted that physical health was important for mental health and that services to support both were equally important. That is, they did not want to lose the emphasis on physical health due to a better focus on mental health since firefighters also have high physical health risks, and both are linked.

“*Healthy body, healthy mind…if we develop a good workout routine, it also alleviates stress, but you work on a healthy body, it’s going to help your mind.*”

The support of fellow firefighter crew members, team leads, and the fire service as a whole were commonly mentioned as a major support system. Social support was highly important to firefighters, and there is a strong sense that other firefighters are the only ones who truly understand the issues they have to deal with. The support from their crew was almost always a positive factor in supporting mental health. Firefighters also mentioned the importance of trusting that management truly valued their mental health and well-being as a priority was also important. Firefighters were encouraged by success stories from firefighters who had recovered from mental illness and hearing their experiences of what helped heal them. Despite the problems that critical event exposures create, some firefighters also acknowledged that seeing the misfortune of others made them develop even greater appreciation for their home and family and that the support of family and friends was often critical in counteracting the exposures from work.

Firefighters acknowledged that firefighter policies and procedures can be helpful and are improving. They had knowledge that many fire services were making efforts to reduce stigma and improve culture, particularly with respect to mental health. The benefits of presumptive legislation that acknowledges that mental health conditions do arise from work exposures and the resulting benefits that arise from that recognition were seen as positive policies. In jurisdictions where presumptive legislation was not in place, timely and equitable management of mental health claims was seen as problematic. Each fire service had its own suite of preventative and treatment programs, and this was also highly variable between fire services in terms of the number and perceived quality of those services.

Firefighters also raised multiple barriers that affected the accessibility, timeliness, or usefulness of mental health supports. They acknowledged that they themselves could be the barrier to accessing help—sometimes because they were unable to identify when they needed help and sometimes because even though they were struggling, they were resistant to seeking help until mental health issues became really big problems. Some of this was attributed to fire service culture. Firefighters acknowledged that the culture in the fire service is changing but still has some drawbacks and that some aspects of culture could be both positive and negative depending on the circumstance or how applied. The very nature of this fire service being grounded in a culture of valuing tradition was seen as respectful but also created a culture of being resistant to change. Firefighters have pride about their strength, but the culture of having to be stronger than everybody else means that you cannot admit weakness or that you do not know something. The culture of dark humour was seen as a helpful way to deal with critical events, but they also acknowledged that sometimes it contributed to a reluctance to admit when you have problems.

Lack of skills in dealing with mental health concerns was raised by multiple firefighters. In some cases, they talked about wanting to help themselves or others but being uncertain about what was the right thing to do. In other cases, they noted that leadership or administrators can have a lack of understanding of mental health. Although firefighters generally reported high levels of adherence to programs, like peer debriefing, some acknowledged that in some cases, there was a lack of adherence and that this should be more consistently implemented.

Multiple sub-themes focused around issues with respect to diversity and the hyper-masculinized environment of the fire services. Both men and women firefighters acknowledged that a masculine-predominant environment had some negative consequences. Some women firefighters reported gender discrimination, and firefighters with other concerns around diversity and inclusion expressed that, in general, the fire culture did not seem to appreciate the importance of diversity and that it could actually be a strength. Women firefighters experienced two sides of the coin in terms of pride in being firefighters and being part of the fire services while also experiencing gender-related hostility or doubts that made them feel less welcome by some of their colleagues, which was detrimental to their mental health.

Many firefighters commented on mental health supports being difficult to access or poorly targeted because the providers were not appropriately aware of the demands of firefighting. Barriers to treatment included that it was often difficult to access, that it could be costly, there were often delays in accessing care even when it was available, that health care providers often did not understand the nature of their work, and that it can be difficult to find the right therapist. Some firefighters who had engaged in therapy acknowledged that establishing a therapeutic alliance with the therapist did not necessarily happen with the first interaction, and sometimes changing therapists was helpful. Although firefighters valued peer support, they did acknowledge that sometimes peer supporters and professionals provided very different approaches or messages. This could be a problem in terms of effectiveness but also in their confidence in the value of either.

With respect to policies and procedures in the fire service, firefighters acknowledged that they were improving but not yet adequate. There were still concerns about stigma around mental health in the fire service. While they valued the increased focus on PTSD, there was also concerns raised that it took away from the broader concerns about mental health as a whole. Thus, some were concerned that the fire service was not addressing the full spectrum of mental health and that substantial gaps remain. Sometimes the concern was lack of preventative training and preparation for what firefighters have to encounter in the field. Sometimes it focused on limited benefits and other times insufficient access to treatment. Again, contextual variation between fire services or municipalities was clear.

Firefighters acknowledged some aspects of dealing with mental health in the workplace can be problematic and have two sides. Although they believed that time away from work may be necessary for mental health, they also acknowledged that there was potential for mental health days to be abused and that there was no way to differentiate needed sick days from unnecessary absenteeism. There were conflicting views about confidentiality. While firefighters were concerned that they had to report to supervisors, which compromised their confidentiality, they also talked about privacy legislation being a barrier to them knowing and helping when people were in trouble.

Table 1 has a summary and representative quotes of the themes and subthemes that arose within barriers and facilitators that are described and inter-linked above.

### 3.3. Firefighters’ Needs

In many ways, the needs expressed by firefighters reflected the symptoms they experienced and the barriers to mental health within the fire service and society as a whole from the themes above.

Discussions focused on training, better prevention and treatment, and improving culture. Some firefighters expressed that training did not adequately prepare them for what was going to be seen in the field, particularly with respect to medical calls. Early resiliency training was commonly endorsed. Firefighters who had gone through resiliency training expressed the need for booster retraining sessions. Training on how to recognize problems with mental health in self or others was seen as a critical component of training.

For departments with less access to programs, this was a major concern, and it was clear that there was substantial variability across fire services with respect to implemented programs. Thus, where mental health programs did not exist, firefighters were particularly interested in resiliency training, peer support, and debriefing to be consistently implemented. Multiple firefighters mentioned the importance of including the family in resiliency training:

“*Should also include families … it was my ex-wife who picked up on all the pieces, but didn’t know what it was.*”

“*Come out with a list of incidents that are recognized as being unanimously traumatic for everyone and have a policy where immediately that crew should be taken out of service. They should have a chance to sit down and talk to someone about it.*”

With respect to treatment, firefighters wanted timely and unlimited therapy and assurances that their services would be confidential. A number mentioned that the claims system was difficult to navigate and that better training and resources for how to appropriately access mental health-related workers’ compensation or benefits was needed. Timely, effective, targeted treatment by professionals who understood the demands and experiences of firefighters was seen as important, and failure to deliver on any of these aspects compromised outcomes:

“*The city needs to hire a psychologist or psychiatrist and get their opinion on how to treat members or firefighters that have mental health issues and get their opinion on how—one of the biggest things is access.*”

A number of firefighters talked about returning to work after a mental health problem and how challenging that process can be for many reasons. It was acknowledged that the critical incidents that can lead to mental health problems cannot be changed, although some firefighters acknowledged that the type of incident that bothered them may be different than their colleagues. For example, repeat drug overdoses, crank calls, etc., were seen as irritants that could have a cumulative negative impact, which was different than the effect of major critical incidents.

Firefighters acknowledged difficulty in controlling exposures since it is impossible to predict what might happen on any given call and that dispatch information gives limited preparation. Further, being selective about which calls you take was not seen as a viable option. For these reasons, although graduated return to work was seen as highly valuable, it was also seen as problematic to implement. Modified work, although valued in the short-term, was not a favoured solution if it involved not doing core firefighting services:

“*The gradual returning to work. You know, starting off with, you know, a few hours of coming into the station without being on the rigs or on the trucks. And then gradually, you know, to a day and then a night, doing a couple of night shifts at work. And then coming back into, you know, being back full time on duty sort of thing.*”

Firefighters agreed that the focus on reducing stigma in the fire service and society at large needs to continue and were concerned about how the public perceives them. Therefore, they thought that public awareness campaigns to ensure the public is aware of their potential problems and that it is no different than any other health problem was often mentioned.

### 3.4. Research Needs

When discussing research needs with firefighters, it was clear that this was an area that they felt uncomfortable in terms of designating specific questions or types of research. While there was strong support for the need for research, firefighters often expressed a lack of knowledge about what specific research should be done. However, four themes emerged as listed below.

1. Awareness and monitoring
  a. Trends in mental health
    i. Document the mental health challenges firefighters experience (goal increase awareness)
    ii. Bi-annual exams/surveys to monitor changes in physical and mental health
2. Understanding mental health
  a. Other mental health issues beyond PTSD
  b. The cumulative effects of mental health exposures
  c. Brain mechanisms that lead to mental health/PTSD issues
  d. Ways to measure exposures and outcomes
3. Better prevention and treatment
  a. Research on early signs and symptoms that a person is in trouble
  b. Design and evaluation of prevention and treatment programs
4. Access to care
  a. Geographic variations in programs/services
  b. Barriers to access to health care services

A number of firefighters mentioned the importance of research that documents mental health concerns and saw this research meeting a variety of needs, including improving awareness about how important a problem mental health is and to be aware of changing trends in mental health. Some firefighters specifically mentioned that (bi)annual screening surveys would be helpful to monitor changes that might occur over time:

“*Research—it can help individuals realise that… the things that we see and do that are negative are going to affect our mental health and that there is nothing, you know, there’s nothing wrong with that.*”

Research priorities around etiology of mental health reflected the lack of understanding of the mechanisms and mediators of mental health. Firefighters mentioned the need to understand other health problems in addition to PTSD, including the cumulative effects of critical event exposures and the brain mechanisms that lead to mental health concerns. A number acknowledged the need for better measurement tools for both exposures and outcomes to understand mental health:

“*We need to understand what happens within our brain and how we can rewire that or have it wired appropriately and what that first 12 h means. So, what the first 24 h means and what we can do about it.*”

“*Death calls, … repetitive nuisance calls that just drive you crazy over a while … the same intoxicated people that you deal with everyday... obviously each call affects everybody else differently. We need to see if there’s actually a pattern to why people are affected or can’t deal with a certain kind of call.*”

“*How do we measure what stigma does to firefighters? How do we measure the culture? How can we measure that had we put in training in place?*”

A number of firefighters suggested research priorities around better prevention and treatment. These included research on the early signs and symptoms that a person is in trouble and design and evaluation of new prevention and treatment programs. A number of firefighters mentioned a research priority around examining geographic variations and barriers to accessing health care services or services across different departments:

“*I guess just to kind of see, you know, the percentage of people who have mental health and maybe just track the treatment and processes that they go through before they return to work to see what’s kind of effective and what’s not.*”

## 4. Discussion

This study provided insights into the mental health experiences, beliefs, needs, and research priorities of Canadian career firefighters. Three overarching themes that arose are the highly variable context across different fire services/regions, uncertainty in mental health, and that for many issues, there are “two sides to the coin”. Although quantitative studies have indicated that mental health concerns are highly prevalent in the fire service [6,7,13,14,42], this study provides more depth on the nature of their symptoms and the impact of those symptoms. A number of the symptoms reported by firefighters are classic symptoms of mental health problems, which is in line with the results of exposure studies [6,7] and screening questionnaires studies that report high rates of mental health symptoms [13,14,42]. Firefighters acknowledged both the use of health services and some maladaptive behaviours that arise directly from those symptoms and the downstream effects that affected family and work. Many of the themes that arose in one area of inquiry were reflected in other areas. For example, the importance of the family was acknowledged as a facilitator and the impact of job stress on the whole family was a concern, which led to an identified need for the family to be involved in resiliency training. The importance of treatment was clear and an identified facilitator, and yet, concerns about it not being sensitive to the unique needs of firefighters, inaccessible, costly, and of unknown effectiveness also occurred across different aspects of our inquiry.

Uncertainty was an overarching latent theme. Firefighters expressed uncertainty as to why some people or some exposures have higher risk, what the processes are for those that need help with mental health problems, what the best thing to do is when someone is struggling, or how policies could be implemented in ways that provide everything that is essential for maintaining firefighter mental health while ensuring that all claims were valid and absences reasonable. While they acknowledge this uncertainty in themselves, it was seen as a barrier that uncertainty exists in management and research knowledge. Therefore, a strong theme in their perceived research needs was better understanding of what factors contribute to mental health problems in ways that would require basic mechanistic studies and real-world observational studies.

Uncertainty was clear with respect to their research priorities. While firefighters were very clear that research was needed, they appeared uncomfortable with making specific suggestions about specific research priorities, which they often attributed to lack of knowledge about research. Despite this uncertainty, themes did emerge defining different types of research that are important for different purposes. Despite the considerable work done to show that there are high rates of mental health concerns in the fire service, ongoing surveillance and continuing to show the importance of mental health concerns was still seen as being relevant. Some firefighters identified measurement issues, which shows they can provide great insights into research priorities despite their lack of confidence in research processes. Prior studies have illustrated that tools designed for the general public do not necessarily function well given the unique context of firefighting [43,44].

Uncertainty also arose due to complexity and the “two sides to the coin” phenomena. Firefighters acknowledged multiple things that could be good in one circumstance and problematic in the other. For example, although peer support was highly valued, some concerns were raised that peer supporters do not have adequate mental health training and may not provide advice on par with what a professional would be able to deliver. Some reported that the content of what peer supporters said conflicted with advice from qualified health professionals, and this added to their uncertainty. Firefighters acknowledged that finding the right therapist was difficult, and there was a great deal of uncertainty about finding the right match or knowing what intervention approach would be effective for their mental health needs. They acknowledged great pride in the fire service’s commitment to tradition, and dark humour was valued; yet these aspects could have a detrimental side as barriers to change and disclosure. This illustrates the difficulty in having prescriptive behavioural norms, policies, or expected outcomes from interventions. It points to the need for flexible, person-centered care that recognizes individuality and complexity. This was further reinforced by the need for greater consideration of diversity in the fire service, which included issues around gender diversity, but also recognizing that all people are diverse and that this should be a source of strength.

The social context and the importance of social support were strong themes that reflect the unique context of firefighting. Firefighters expressed the importance of their firefighter family, their crew, and the larger fire service, which included feelings that other people (families, health care workers, the public) could not possibly understand the issues they faced. This increased the importance of their social connectedness to their firefighter family. Letting down their crew was a major concern about the consequences of mental health challenges. Thus, while they acknowledged that graduated return to work was important, they also felt that management did not always understand how disruptive it was to move them to a different crew because that could take away the social support that was helping their mental health and functioning. While firefighters have great pride in the work that they do, the culture of the fire service and the common bonds that they share with other firefighters, they also acknowledged that some aspects of hyper-masculine environments could be detrimental in terms of the stigma associated with mental health concerns, the acceptance for diverse people, and the responses to disclosure of mental health challenges.

There was frequent acknowledgement of progress in the fire service and the substantial investments made by government, employers, management, and health professionals to better understand and address their mental health needs. However, there was also a strong thread across all the areas of discussion that there is much room for improvement. In listening to different firefighters across different services, it was clear that there is substantial inequality in the availability of services across different locations, which is partially related to geography, local politics, socioeconomic factors, the role of champions, and historical factors. These contextual factors mean that mental health policies and programs should be informed by local contextual data and priorities. However, systemic issues, like the hyper-masculine work environment and societal bias against mental health, interplay with local contextual factors, and the broader needs for equity, diversity, and inclusion can be magnified within the fire service.

## 5. Conclusions

In summary, this work reflected and described the complex and variable personal, fire service, and societal contextual factors that influence how critical events in the fire service are managed across different fire services and the profound impact of mental health concerns that extend to the firefighters, their family, and the fire service. Better access to services that are timely, individualized, evidence-informed, and that recognize that finding the right therapeutic relationship can take time and be variable for different firefighters is needed. Despite uncertainty in what is the right thing to do, the need for action is clear. A priority is a need to develop greater capacity and appropriateness in health care services that recognize and meet the unique needs of firefighters since access and appropriateness of care were strong themes, and prevention cannot be expected to eliminate the need for care. The larger societal issues with lack of appropriate mental health care are reflected in the gaps left for firefighters who have elevated needs for these services. Firefighters, management, and clinicians are likely to be challenged by the lack of certainty and evidence to inform the best management of mental health conditions, and firefighters agree on the need for research to improve this situation.

## Figures and Tables

**Table 1:** Barriers and Facilitators Themes Identified by Firefighters, with selected illustrative quotes.

	Facilitators	Barriers
**Personal**	a. Seeing misfortune of others helps you appreciate your home and family more and that you are lucky to have them b. Physical health supports mental health c. Using services when needed	a. Competing demands of employment b. Resistance to seeking care c. Embarrassment in disclosure to colleagues
**Quotes**	*You can go to some pretty tough calls, and it just makes you appreciate the little things that you have in life. You know, your healthy children, healthy parents. It, kind of, can open your eyes and... I mean, the things that stress you out or you’re not too happy about, you know, life isn’t that bad. It, kind of, makes you appreciate everything that’s around you.*	b. *People don’t understand that the cure could be ten feet away, but you don’t feel like getting up to walk over ten feet to get it.* c. *I (am) going to talk to someone that may be working on the truck with them, or on another truck that they run into, and that—you know, I mean, I think it’s all in good intention, but just the fact that it’s someone that you work with, or could run into at work, I think people hesitate to talk to people.*
**Firefighter Work and Culture**	a. Efforts to reduce stigma are improving culture b. Attention to mental health is improving	a. Nature of work b. Culture of tradition in fire service can mean being resistant to change c. Culture of strength and needing to be there means you cannot admit when you do not know something d. Culture: we have to be stronger than everybody else e. Stigma f. Lack of appreciation that humans are diverse... and diversity can be a strength g. Culture of dark humor and teasing helpful in some ways but other times increases reluctance to admit to problems h. Hypermasculinity i. Gender discrimination
**Quotes**	*There’s been a negative stigma for years around mental health, but with this shift, it seems that within the past couple of years even, guys are able to talk about it more openly*	a. *We’ve seen—we see a lot of deaths, you know. My 20 years, I’ve got at least 50 people that have died in my presence or before I got there and some horrifically, you know. You can’t unsee some stuff. It would be great if you can unsee it. But you’ve seen it, and I don’t see how we’re going to change that. It’s our job. Unfortunately, is not one that everybody can do, and it’s not one that’s always very pleasant. So, I don’t think—I think that’s the biggest challenge is—is like war. You can’t—you can’t filter the stuff that we see.* *(I) consider myself to be a pretty strong personality, and strong person, but, like I say, after 22 years, it has gotten to me. So maybe just, you know, let people know what the job really is. It’s not just firefighting.* c. *We don’t need help, we are the helpers, we’re the fixers, and we don’t need to help but you know, that may be a barrier that we have to get through and prove to these guys that, “Hey, okay, you’ve got this Mr. Firefighter attitude you know, tough guy. But really, we’re all human, and we’re going to see some things on different calls that might affect different guys in different ways.* e. *The stigma we are beating down, but it still exists heavily, and for a firefighter to come out and say that they’re suffering and/or having challenges with, you know, the work that they’re doing and some of the mental health issues around it is still really difficult for them to do it, and they literally suffer in silence.* g. *It’s almost, like black humour that we have, you know? It’s kind of dark humour, but it seems to work for us, on the whole. I mean, it may not work for everybody.* *If I hear the expression suck-it-up buttercup one more time…* h. *The fire service is a very—it’s a gendered occupation, typically, you know, male-populated, so that whole sort of chauvinistic, tough guy culture.* i. *I think it’s all just this idea (of women) being soft. We only have two women on the floor right now. And I’m one of them. And there’s still all these kind of comments, if you can’t do something... it(‘s) because you have ovaries or something like that.*
**Firefighter-specific training and programs**	a. Awareness of things in place b. Peer support c. Resiliency training d. Mental health policies e. Critical incident response f. Suicidal awareness programs g. PTSD prevention plan h. Road to Mental Readiness or Resilient Minds^TM^ i. Comprehensive physical and mental health monitoring	a. Addressing part of the spectrum of detection and treatment but leaving gaps in others b. Peer support may not provide same approach message or effectiveness as professional support c. Challenges in gradual return to work d. Insufficient firefighter training for medical roles e. Lack of programs in some services f. Lack of similar training for certain roles can lead to uneven distribution of stressors on team
**Quotes**	a/b. *Our department has a lot of access to it, is actually very good. We have a family—employee family assistance programme. We have counsellors; if you so choose to engage them, they will help you. We have a peer-driven critical incident stress management team. Our administration has scheduled multiple training—mandatory training options for suicide, Road to Mental Readiness, and continue to add in more mental health training. The municipality has all sorts of procedures in place for it.* c. *We’re putting ourselves in hazardous situations, and there’s a lot of studies... indicate that we’re going to be vulnerable to...cancer, respiratory diseases, and stuff like that. but at least if you’re getting regular proper medicals specifically to identify certain things,... I think some of those things might help your mental state.*	a. *The preventative side of the mental health aspect isn’t quite there yet. We have more of the reactive side to it.* d. *… only really got into the medical end in 2010... and that completely changes the job for firefighters. I’ve seen a lot more people that have died in my arms on a medical call doing CPR than I’ve ever seen die in a fire... when you see them in a fire they’re burned, they don’t look like a person anymore, that human contact isn’t there. It’s an object. So, the impact isn’t the same as showing up in someone’s house, and someone’s talking to you, and their eyes roll back in his head, and then they die in front of you, and the family starts yelling at you to do something. I mean that’s totally a different impact, and we were never trained for that.* e. *There’s very little to none. The only thing that exists in (large city name) is our employee assistance program. But an employee assistance program that was, you know, brought up over the years to deal with gambling problems, drinking problems, and divorce. It was never brought up to deal with PTSD. So, we have no formal policy, no formal training. No formal directives, either there’s no mandatory debriefing, there’s not mandatory consultation with a psychologist to really deal with what happens, and then often when it does happen, the employer and Workman’s Compensation even can deny. All we get back is that you’re a firefighter, seeing dead people is normal. Suck it up or change your job.* f. *We’re not all obliged to be cross trained in medical. It’s voluntary. So, you could have four guys on a truck, and you only need two that can do the medical calls. But if you’re only two, you’re constantly doing it, there’s no rotation, there’s no break you never get from it.*
**Firefighter Leadership/** **Management**	a. Trust employer that they are generally concerned about your mental health and well-being b. Follow-up/checking in with people for delayed responses c. Cross checks in the system as to who can bring in mental health supports—by firefighter request or supervisory assessed need	a. Lack of administrators/leadership understanding of PTSD/mental health b. Lack of adherence to processes like peer debriefing c. Real or perceived potential for mental health days to be abused d. Need to report issues to a supervisor—loss of confidentiality e. Unclear claims process
**Quotes**	b. *It could only be a month after a call, to say hey, like do you still... that call, are you doing okay with that call?*	a. *What I’ve been faced with is even with our administration, even head of HR, by posing this question to them, I said to our chief, I said okay, because he doesn’t believe me, I said okay, in five minutes I want you to do a presentation on PTSD, and you can’t Google it. His face dropped. He couldn’t do it, but (n)either can either one of our deputies, (n)either can head of HR.* *My recommendation is going to a health care professional and get their opinion. The people at the WSIB are not psychologists and psychiatrists. The girl working for the return-to-work for the city is not a psychologist or psychiatrist. Neither of them has degrees in medicine.* e. *I would like to see is much more clarity and much more guidance on how to file a mental health claim.*
**Social Supports**	The support of fellow firefighters Success stories from people who recovered from mental illness and talking about what helped heal them Social support from family and friends	It’s not always clear how to help someone Isolation Loss of team
**Quotes**	c. *I think having a strong support of your family, friends, coworkers, … I think it’s all going to contribute to reducing the mental health issues.*	*You know, we’ve had those conversations, and I just, you know, one of my—one of my good friends just cut his brachial artery on purpose. I could kind of see that one coming, and that’s what makes me angry is you know, everyone sees this thing coming, but why did it come?probably the hardest part in dealing with mental health and stressors is not knowing how to help or where to start.* *If you’re going home to an empty apartment at—in the middle of the night, after a bad call, well, then your mind starts to wonder, and probably that’s when you end up in some dark places.* *Working with a good crew is very important, and unfortunately, our city fails to recognize the importance of keeping crews together and letting them build like a family and be able to talk about their problems.*
**Health care**	Ability to coordinate with health care professionals Timely access to care Professionals who “get” firefighters	Doctors manage process but don’t have RTW options for mental health, no specific restrictions for mental health Care that is difficult to access Finding the right therapist who can help Cost of treatment Treatment delays Health care providers who do not understand job demands Challenges in diagnosis
**Quotes**		b. *Or sometimes you’ll be given a phone number or whatever, and it’s like jumping through hoops trying to get the help you need, and so it’s a more humiliating experience than it is enlightening or helpful.* *You know, you can get 100 GPs, but try to find one psychiatrist or psychologist that, you know, you can get a—get into within the current month or even the current three months is pretty difficult.* c. *Went through EAP a number of times, probably eight times in one year … the therapist that I was connecting with, they weren’t good connects for me, so I was believing that I was the problem. It’s really important for us to get to the best care possible, and right now, I see roadblocks there, and it took me five years, actually, to get to a place where I got the right therapist in place, and that five years was very, very dark, and dangerous places for me.* d. *My $500 cap on my insurance is over. I can’t afford any more help.* e. *Would be kind of nice if somebody did need help, and they kind of wanted it right away that they didn’t have to wait you know like a week or a couple of weeks just to access it … It delays everything. And not only does it delay them getting the help that they need, but it kind of sets everything back, and it could make their problem a little bit worse because they’re not getting the help when they need it.* f. *The system is there, but like I said, what I wish was there is to have a doctor who can help us, by understanding our line of work. That is the most important thing. The same thing as if I got an astronaut ask me to help him to work on the space shuttle. I can help him, but I don’t understand him.* *EAP, with the city of (name withheld) is absolutely unworthy. They do not understand firefighting and the culture around it. We’ve heard some horror stories about that. So, it seems to be more that people are left looking for their own resources.* g. *We don’t take someone’s necessary word for it, but at the same time, we’re aware that it’s not that we don’t believe them, but self-diagnosis on it is not necessarily the answer.*
**Societal attitudes and policies**	Efforts to reduce stigma are improving culture and attention to mental health Presumptive legislation	Focus on PTSD can take away the need for broad concern on mental health Privacy legislation means that we often don’t know when someone’s off because they’re in trouble Stigma Lack of understanding of mental health Expectations of firefighters Lack of policies for mental health
**Quotes**	*The stigma, we are beating it down.* *Now with presumptive legislation, that’s getting much better.*	c. *There is still a misunderstanding that it’s okay to have a broken wrist, but it’s not okay to have a sick or injured psychological mind.* *…. the rest of the world … see mental health injuries as a weakness whereas it’s absolutely not.* d. *And it doesn’t matter whether it’s gruesome or not. I’ve had people—you know, arms—legs and arms in my hand that weren’t attached to them, and they were still alive. Didn’t bother me at all. It’s kind of furniture. But every once in a while, something gets under you or past you that sticks with your memory. And I don’t—I can’t explain it, I don’t know how it affected me ultimately in the end. I’m not saying it didn’t, I just don’t know how it did.* e. *One of things that I learned throughout my career is that the model of who we are as firefighters, they actually created it in children’s books, and how do I ever live up to that?* f. *Since post-traumatic stress disorder injuries isn’t a recognized work illness in Quebec, it’s more of a regular sick leave. It’s the same as if you’ve been off with a really bad cold or been off with anything else. So, the return to work is very much the same. You don’t come back to work until your doctor says you can come back to work. And when he says that you can, you come back to work. Because there’s no policy or procedure, there’s no difference.*

## Data Availability

The data are not publicly available due to ethical and privacy restrictions.

## Supplementary Materials

The following are available online at https://www.mdpi.com/article/10.3390/ijerph182312666/s1, File S1: Semi-structured interview guide.
