# Supplementary material for: Canadian Career Firefighters’ Mental Health Impacts and Priorities
Source: Int J Environ Res Public Health. 2021 Dec 1;18(23):12666. doi: 10.3390/ijerph182312666 (PMC8656638; doi:10.3390/ijerph182312666)
Supplement: Supplementary file 1 [file ijerph-18-12666-s001.zip › ijerph-1468316-supplementary.pdf]

Supplementary File 1: Semi-structured interview Guide

1. How do you think stress and mental health injuries affect firefighters and their families?
2. How do you think stress and mental health injuries affect firefighting?
3. What access does your team have to mental health supports (like education, mental health strategies, peer support, professional supports, or return to work)?
4. What is your employer's return-to-work process for firefighters who are on leave due to stress or mental health injuries?
5. What are the facilitators or things that can help an injured firefighter's return to firefighting?
6. What are the barriers or things that can hinder an injured firefighter's return to firefighting?
7. What types of things (i.e., interventions, tools, approaches) do you wish existed to help RTW situations around stress and mental health injuries?
8. What kinds of things do you think would help to prevent firefighters' mental health injuries?
9. What are some of the challenges in working to prevent firefighters' mental health injuries? Why may it be difficult?
10. What types of things do you wish existed to help improve firefighters' mental health?
11. How can research be used to help improve firefighters' mental health?
